# The traditional herb *Polygonum hydropiper* from China: a comprehensive review on phytochemistry, pharmacological activities and applications

**DOI:** 10.1080/13880209.2023.2208639

**Published:** 2023-05-17

**Authors:** Yi-Dan Kong, Ying Qi, Na Cui, Zhi-Hong Zhang, Na Wei, Chang-Fu Wang, Yuan-Ning Zeng, Yan-Ping Sun, Hai-Xue Kuang, Qiu-Hong Wang

**Affiliations:** a Key Laboratory of Chinese Materia Medica, Ministry of Education, Heilongjiang University of Chinese Medicine, Harbin, China; b Guangdong Engineering Technology Research Center for Standardized Processing of Chinese Materia Medica, School of Chinese Materia Media, Guangdong Pharmaceutical University, Guangzhou, China; c School of Pharmacy, Hainan Medical University, Haikou, China

**Keywords:** Flavonoids, volatile oils, antibacterial and antifungal effects, antifeedant and insecticidal effects, traditional and modern clinical applications

## Abstract

**Context:**

*Polygonum hydropiper* L. (Polygonaceae) (PH) is a traditional Chinese traditional medicine with a pungent flavor and mild drug properties. PH is mainly distributed in the channel tropism in the stomach and large intestine. PH has multiple uses and can be used to treat a variety of diseases for a long time.

**Objective:**

This review summarizes the phytochemical and pharmacological activities, and applications of PH from 1980 to 2022. We also provide suggestions for promoting further research and developing additional applications of PH.

**Methods:**

The data and information on PH from 1980 to 2022 reviewed in this article were obtained from scientific databases, including Science Direct, PubMed, Science Citation Index, SciFinder Scholar (SciFinder), Springer, American Chemical Society (ACS) Publications, and China National Knowledge Infrastructure (CNKI), etc. Some information was obtained from classic literature on traditional Chinese medicines. The search terms were *Polygonum hydropiper*, phytochemistry compositions of *Polygonum hydropiper*, pharmacological activities of *Polygonum hydropiper*, and applications of *Polygonum hydropiper*.

**Results:**

The comprehensive analysis of the literature resulted in 324 compounds being isolated, identified, and reported from PH. Regarding traditional uses, the majority of phytochemical and pharmacological studies have indicated the diverse bioactivities of PH extracts, flavonoids, and volatile oil elements, including antibacterial, antifungal, insecticidal, antioxidant, and anti-inflammatory.

**Conclusions:**

PH has a long history of diversified medicinal uses, some of which have been verified in modern pharmacological studies. Further detailed studies are required to establish scientific and reasonable quality evaluation standards and action mechanisms of active constituents from PH.

## Introduction

The Polygonaceae family consists of 50 genera and 1120 species; of these, approximately 13 genera and 238 species are found in China. *Polygonum* is the major Polygonaceae genus for medicinal purposes, with approximately 300 species worldwide. In total, there are 131 species and 31 varieties of *Polygonum* in China; 72 species have medicinal value, are distributed in many provinces in northern and southern China, and are commonly found on roadsides or in watery wet places (Wang et al. 1996; Wang 2008; Wang et al. 2012).

*Polygonum hydropiper* (PH) is the whole plant and stem of *Polygonum hydropiper* L., an annual herb (Wang 1996). PH was first recorded in *Tang Materia Medica* (Cheng et al. 1999; Li et al. 2003), and the 1977 Edition of the Chinese Pharmacopoeia included the whole herb of PH. The leaves of PH have deep red spots, which are exclusive characteristics of PH and are distinguished from other Polygonaceae plants. PH, known as laliao in Chinese, is widely used as a traditional herbal medicine. Among the Chinese people, PH is also called shuiliao, liaoyacai, liuliao, laliaocao, liaozicao, banjiaocao, litongcao, etc. (Wang, Liu et al. 1996; Wang 2014). PH often grows in patches in low mountain areas, hills, plain hillsides, riverbanks, and other moist places. PH is widely distributed in the northern and southern provinces of China, including Hebei, Henan, Shanxi, Jiangsu, Zhejiang, Hubei, Fujian, Jiangxi, Guangdong, Guangxi, and Yunnan (Zhang 2004). According to the theory of traditional Chinese medicine, PH has a pungent flavor and mild drug properties. The meridian tropism of PH is the stomach and large intestine. PH has many traditional effects, such as dampness-resolving, stagnation-removing, wind-expelling and detumescence; it is mainly used to treat and relieve some diseases including dysentery, enter gastritis, diarrhea, dermatophytosis beriberi, itch, rheumatoid arthritis, hemostasis swelling pain, and functional uterine hemorrhage. Topical treatment includes snakebite and skin eczema, etc. (Li 2017). At present, PH is often used in combination with other traditional Chinese medicines in the clinic to treat rheumatism, traumatic injury, skin diseases, acute and chronic gastroenteritis, chronic rhinitis, gynecological diseases, ophthalmological diseases, and sebaceous cysts, etc. (Huang and Zhen 2013).

Previous studies in the 1990s found that PH has antimicrobial, insecticidal, antioxidant, antitumor, and other biological activities (Haraguchi et al. 1992, 1993, 1996; Yagi et al. 1994). The active ingredients are flavonoids and volatile oils. In recent years, there have been many reports about the phytochemical composition and pharmacological activities of PH worldwide. Flavonoids have antimicrobial and anti-inflammatory effects, and volatile terpenoids have insecticidal activities, which can provide some references for the development of new drugs and new plant-based pesticides. Moreover, there is a certain development prospect in the fields of medicine, food, botanical pesticides, veterinary medicine, and food additives (Zeng et al. 2006; Huang and Zhen 2013). However, an overall review of these factors is lacking. In this review, the phytochemical constituents, pharmacological activities, and applications of PH have been summarized for the further detailed research, with the hope that PH can be fully and effectively developed and utilized.

## Methods

This literature review of PH covers the reports from 1980 to 2022. The data and information were obtained from multiple scientific databases, including Science Direct, PubMed, Science Citation Index, Sci Finder, Springer, ACS Publications, Innojoy, Google Scholar, Baidu Scholar, and China National Knowledge Infrastructure (CNKI). Additional information was obtained from classic literature on traditional Chinese medicines, as well as PhD and MSc theses in the school library, and downloaded from CNKI, read manually and analyzed in groups. The search terms were *Polygonum hydropiper*, phytochemistry compositions of *Polygonum hydropiper*, pharmacological activities of *Polygonum hydropiper*, applications of *Polygonum hydropiper* and other related search terms. We excluded reports and articles that appeared in some news media or newspapers, as well as literature that was not published in formal professional magazines or periodicals.

### Phytochemistry

**Figure 1.**
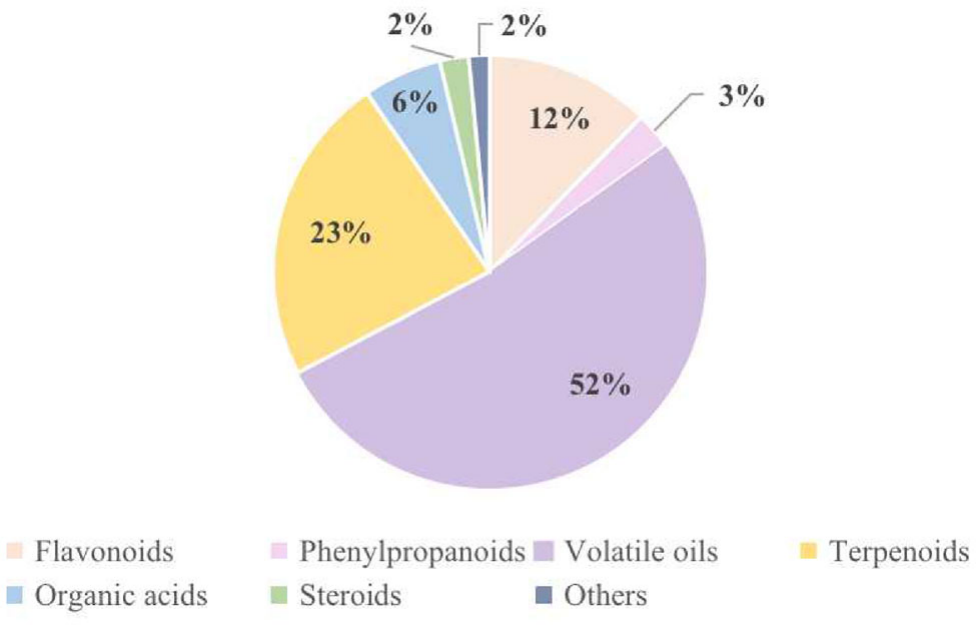
The percentage of chemical constituents.

To date, 324 compounds have been isolated and identified from PH, and researchers have adopted multiple separation techniques for the isolation and purification of chemical constituents from PH, including 40 flavonoids, 9 phenylpropanoids, 169 volatile oils, 75 terpenoids, 19 organic acids, 7 steroids, and 5 others. The percentages of chemical constituents are shown in Figure 1.

### Flavonoids

**Figure 2.**
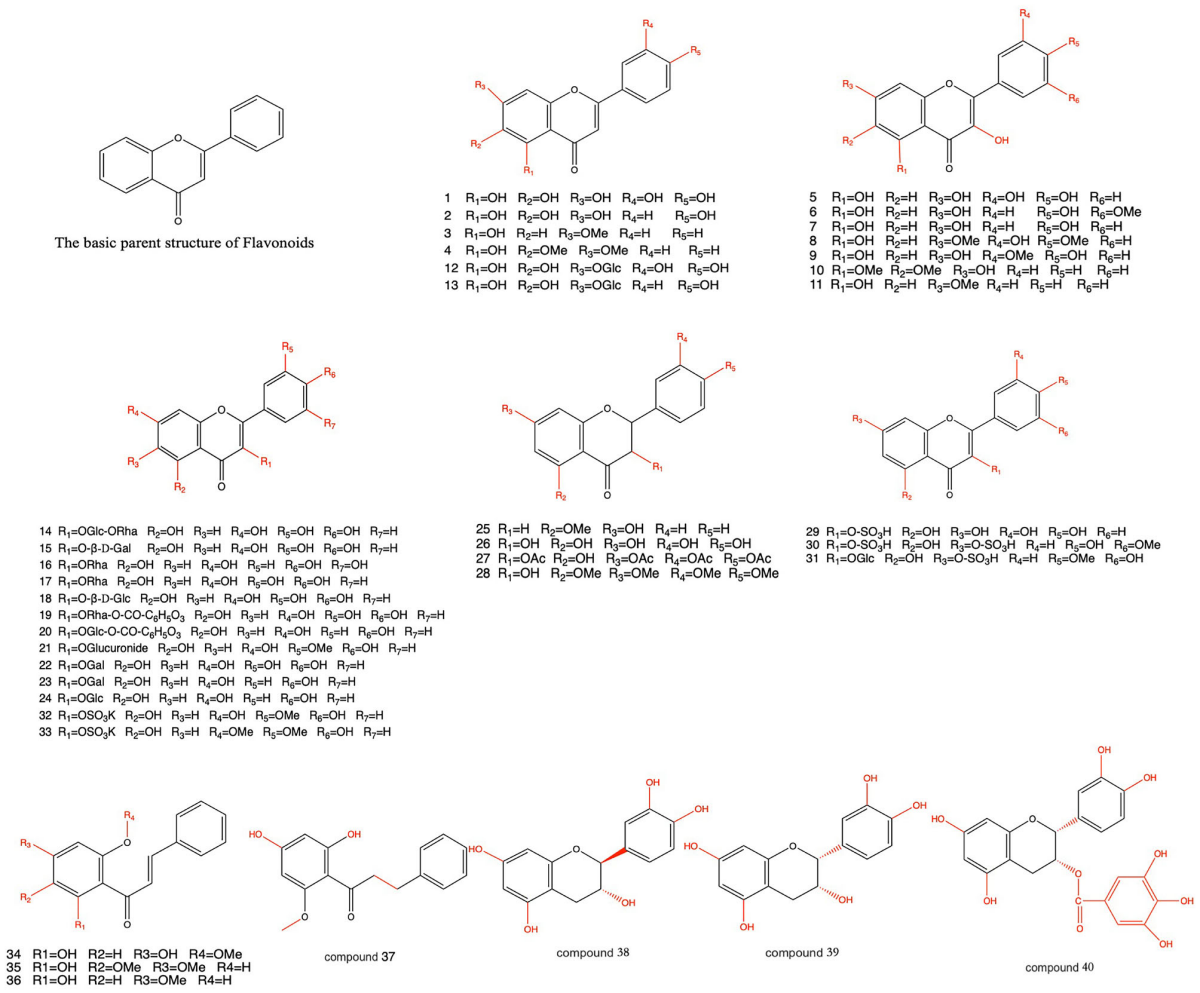
Structures of Compounds 1 to 40.

Flavonoid ingredients are the main active ingredients of PH. In addition, flavonoid glycosides, flavonol and its glycosides, flavone and its glycosides, chalcone, and dihydrochalcone are found in PH. The species, compounds, and molecular formulas of PH are shown in Table 1. The basic parent nuclei and structures of flavonoids **1** to **40** are shown in Figure 2.

**Table 1.** The species, compounds, and molecular formulas of flavonoids.

No.	Species		Compound	Ref.
1	Flavonoid		6-hydroxyluteolin	Zhao et al. 2003
2	Flavonoid		scutillarein aglycone	Zhao et al. 2003
3	Flavonoid		pinostrobin	Zhao et al. 2003
4	Flavonoid		onysilin	Zhao et al. 2003
5	Flavonol		quercetin	Zhao et al. 2003
6	Flavonol		isorhamnetin	He et al. 2014; Xiao et al. 2018
7	Flavonol		kaempferol	Xiao et al. 2018
8	Flavonol		7,4′-dimethylquercetin	Haraguchi et al. 1992
9	Flavonol		3′-methylquercetin	Haraguchi et al. 1992
10	Flavonol		3,7-dihydroxy-5,6-dimethoxy-flavone	Xiao 2018
11	Flavonol		isalpin (3,5-dihydroxy-7-methoxy-flavone)	Xiao 2018
12	Flavonoid glycoside		6-hydroxyluteolin	Xiao 2018
13	Flavonoid glycoside		7-*O*-β-d-glucopyranoside	Zhao et al. 2003
14	Flavonol glycosides		rutin	Xiao 2018
15	Flavonol glycosides		hyperoside	Xiao 2018
16	Flavonol glycosides		quercitrin	Xiao 2018
17	Flavonol glycosides		quercetin-3-*O*-rhamnoside	Wang et al. 2018
18	Flavonol glycosides		isoquercitrin	Wang et al. 2018
19	Flavonol glycosides		galloyl quercitrin	Zhao et al. 2003
20	Flavonol glycosides		galloyl kaempferol 3-glucoside	Zhao et al. 2003
21	Flavonol glycosides		quercetin 3-*O*-β-d-glucuronide	Zhao et al. 2003
22	Flavonol glycosides		quercetin-3-*O*-β-galactoside	Li et al. 2017
23	Flavonol glycosides		kaempferol-3-*O*-β-galactoside	Li et al. 2017
24	Flavonol glycosides		kaempferol-3-*O*-glucopyranoside	Wang et al. 2018
25	Dihydroflavone		7-hydroxy-5-methoxy-flavanone	Wang et al. 2018
26	Dihydroflavone		(2*R*,3*R*)-(+) taxifolin	Miyazawa and Tamura 2007
27	Dihydroflavone		3,7,3′,4′-taxifolin tetraacetate	Miyazawa and Tamura 2007
28	Dihydroflavone	5,7,3′,4′-taxifolin tetrametyl ether		Miyazawa and Tamura 2007
29	Flavonol sulfate	quercetin-3-sulphate		Yagi et al. 1994
30	Flavonol sulfate	isorhamnetin-3,7-disulpgate		Yagi et al. 1994
31	Flavonol sulfate	tamarixetin-3-glucoside-7-sulpgate		Yagi et al. 1994
32	Flavonol sulfate	percicarin		Haraguchi et al. 1996
33	Flavonol sulfate	rhamnazin-3-sulfate		Haraguchi et al. 1996
34	Chalcone	cardamomin (2′,4′-dihydroxy-6′-methoxychalcone)		Xiao 2018; Wang et al. 2018
35	Chalcone	2′,6′-dihydroxy-3′,4′-dimethoxychalcone		Kurkina et al. 2013
36	Chalcone	polygochalcone		Kurkina et al. 2013
37	Dihydrochalcone	uvangoletin		Wang et al. 2018
38	Flavanol	catechin		Ono et al. 1998
39	Flavanol	epicatechin		Ono et al. 1998
40	Flavanol	epicatechin-3-*O*-gallate		Ono et al. 1998

### Phenylpropanoids

**Figure 3.**
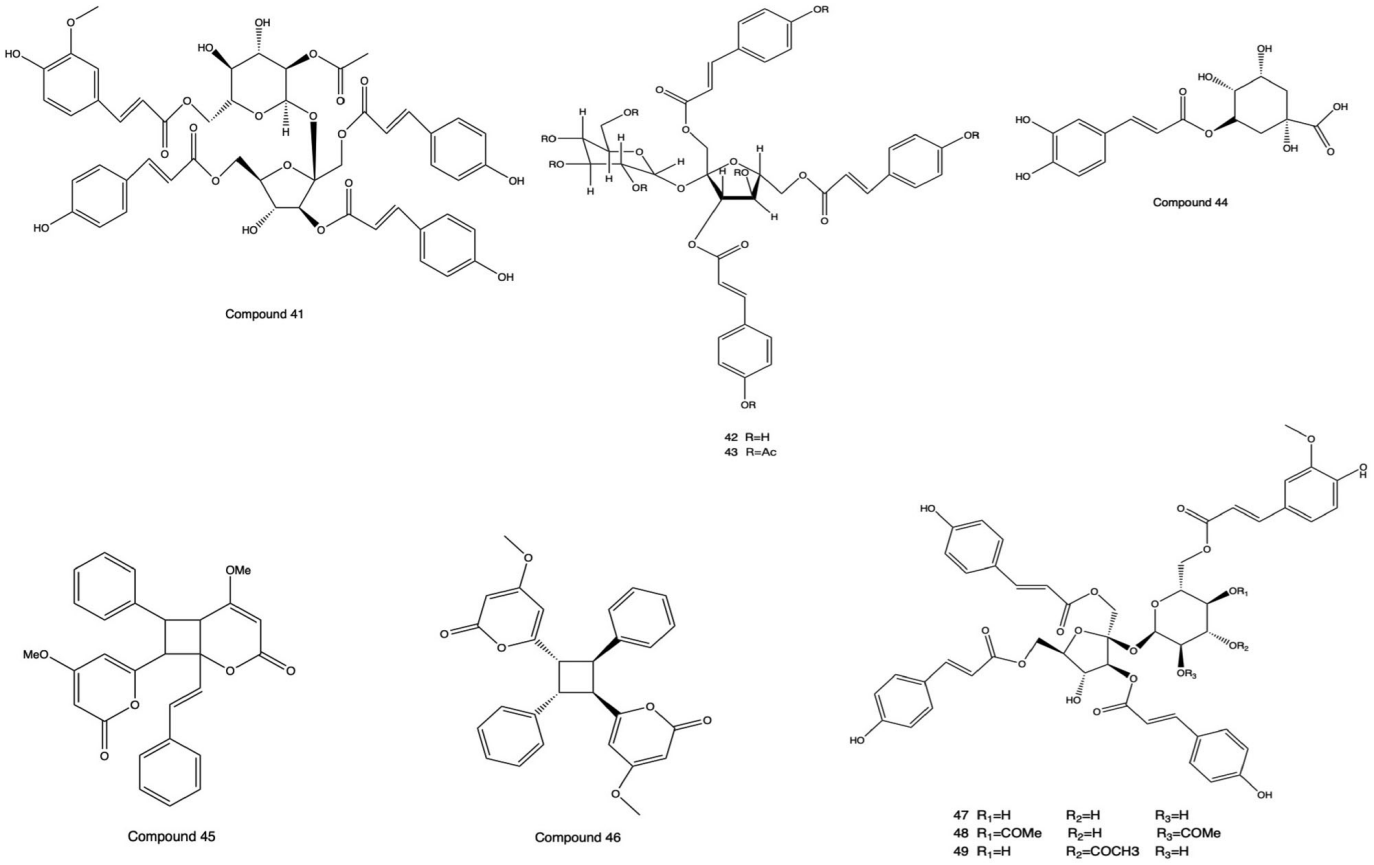
Structures of Compounds **41** to **49**.

Most of the phenylpropanoids contained in PH are simple phenylpropanoids and coumarins. Vanicoside A′ (**41**), hydropiperoside B (**42**), and hydropiperoside A (**43**) were isolated, purified and distinguished from PH by column chromatography with silica gel and Sephadex, preparative high performance liquid chromatography (pre-HPLC), NMR, and ESI-MS (Fukuyama et al. 1983; Kiem et al. 2008; Wang et al. 2018). Chlorogenic acid (**44**) was separated and identified from PH by chromatography with silica gel and MCI, physicochemical properties, and spectral analysis for the first time (Xu et al. 2017). Aniba-dimer A (**45**) and 6,6′-((1 *R*,2*R*,3*S*,4*S*)-2,4-diphenylcyclobutane-1,3-diyl) *bis* (4-methoxy-2H-pyran-2-one) (**46**) were separated from the dichloromethane part of PHL (Xiao 2018). Vanicoside B (**47**), vanicoside E (**48**) and vanicoside F (**49**) were isolated from the dichloromethane-soluble portion of the ethanol extract of PH (Xiao et al. 2017). The structures of phenylpropanoids from **41** to **49** are shown in Figure 3.

### Volatile oils

The volatile oils stored in PH are extremely complex, mainly sesquiterpenoids, and contain enol tautomerisms and sterols. Due to the volatility and instability of the volatile oils, different origins, different months, and different extraction methods can affect the volatile components of PH.

**Figure 4.**
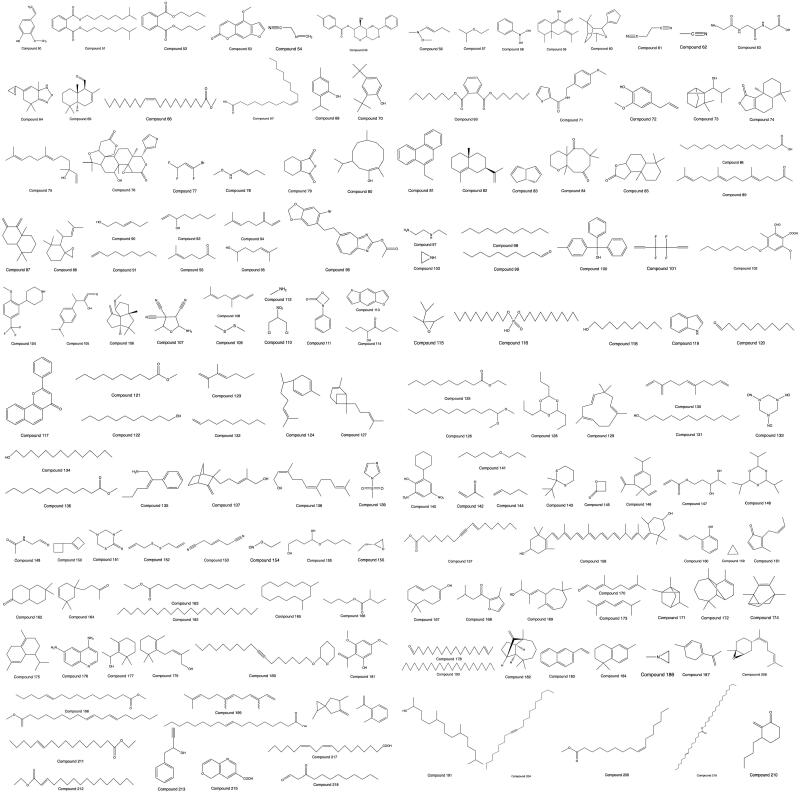
Structures of Compounds **50** to **218**.

Eight volatile oil components were first separated and identified by the supercritical CO_2_ technique and combined GC–MS coupling technique (Zhang and Zeng 2005). A total of 125 volatile oils from the aboveground parts of PH grown in May, August, and November in Guizhou in 2018 were obtained and identified by steam distillation and GC–MS coupling techniques; they had 15 similar components (Table 2) (Yu et al. 2018). Volatile oils of PH were first extracted by the methods of steam distillation and CO_2_ supercritical extraction, and identified by the GC–MS coupling technique, which yielded 14 and 4 volatile oil-like components, respectively, including 2 similar compounds (Li 2007). Seventy-five volatile components from PH in Guizhou contained the same 5 compounds, and they were obtained and distinguished by solid phase extraction, steam distillation and GC–MS coupling techniques (Lin et al. 2012). Fifty-three volatile components of PH from Hunan were obtained by steam distillation combined with GC–MS (Yao et al. 1999). Six volatile components of PH were first found first from an ether extraction part of alcohol extraction by GC–MS (Zeng 2007). Altogether, 103 volatile components of PH were isolated and identified by steam distillation and GC–MS coupling techniques (Wu et al. 2007; Liu, Zhang et al. 2009; Wang et al. 2017). The volatile oil compounds (from **50** to **218**) of PH are shown in Table 2. The structures of phenylpropanoids **50** to **218** are shown in Figure 4.

**Table 2.** Compounds of volatile oils.

No.	Compound	Ref.	No.	Compound	Ref.
**50**	4-vinyl-2-methoxy-phenol	Yao et al. 1999	**66**	11-eicosenoic acid methyl ester	Yao et al. 1999
**51**	di-*iso*-nonylphtalate	Zeng et al. 2007	**67**	oleic acid	Yao et al. 1999
**52**	dibutyl phthalate	Zeng et al. 2007	**68**	thymol	Zeng et al. 2007
**53**	bergapten	Yu et al. 2018	**69**	diheptyl phthalate	Zeng et al. 2007
**54**	2-methylenimine acetonitrile	Liu, Zhang et al. 2009	**70**	2,4-di-*tert*-butylphenyl	Zeng et al. 2007
**55**	1,5-anhydro-4,6-*O*-benzylidene-2,3-dideoxy-d-erythohexlenitol	Zhang and Zeng 2005	**71**	*N*-(4-methoxyphenyl)-2-thiophene carboxamide	Liu, Zhang et al. 2009
**56**	*N*-butene-*N*-oxide methotrexate	Liu, Zhang et al. 2009	**72**	4-*allyl*-2-methoxyphenol	Zeng et al. 2007
**57**	ethyl isopropyl ether	Liu, Zhang et al. 2009	**73**	8-(3-methyl-2-butenyl)-tricyclene	Zhang and Zeng 2005
**58**	bisabolene	Yu et al. 2018; Lin et al. 2012; Yao et al. 1999	**74**	naphthol-[1,2-*c*]-furan-1(3H)-one,5,5a,6,7,8,9,9b-octahydro-6,6, 9a-trimethyl-[5a*S*-(5a,9ab,9ba)]-(-)-drimenin	Lin et al. 2012
**59**	1-hydroxy-4a,5-dimethyl-3-(propan-2-ylidene)-4,4a,5, 6-tetrahydronaphthalen-2(3H)-one	Yu et al. 2018	**75**	nerolidol	Yu et al. 2018; Lin et al. 2012; Yao et al. 1999
**60**	4,6,6-trimethyl-2-(3-methyl-1,3-cyclovinyl)-3-oxatricyclo-[5.1.0 (2,4)]-octane	Li 2007	**76**	bergamotol	Yu et al. 2018; Lin et al. 2012; Yao et al. 1999
**61**	succinonitrile	Liu, Zhang et al. 2009	**77**	1-beomo-2,3,3,-trifluoro-1-propene	Liu, Zhang et al. 2009
**62**	acetonitrile	Liu, Zhang et al. 2009	**78**	*N*-butylene-*N*-oxide methylamine	Liu, Zhang et al. 2009
**63**	acetylamino acetaldehy	Liu, Zhang et al. 2009	**79**	3,4,5,6-tetrahydrophthalic anhydride	Liu, Zhang et al. 2009
**64**	2H-cyclopropa-[*g*]-benzofuran,4,4,6b-trimethyl-2-(1-methylethenyl)	Yu et al. 2018	**80**	(1*R*,7*S*,*E*)-7-isopropyl-4,10-dimethylenecyclodec-5-enol	Yu et al. 2018
**65**	drim-7-en-11-ol	Yu et al. 2018	**81**	9-ethylphenanthrene	Liu, Zhang et al. 2009
**82**	2-((2S,4aR)-4a,8-dimethyl-1,2,3,4,4a,5,6,7-octahydronaphthalen-2-yl)-propan	Yu et al. 2018	**100**	benzenemethanol	Yu et al. 2018; Yao et al. 1999
**83**	1,2,3,3a,4,6a-6H-cyclopentadiene	Liu, Zhang et al. 2009	**101**	3,3,4,4-tetrafluoro-1,5-hexadiene	Liu, Zhang et al. 2009
**84**	1,5,5,8-tetramethyl-12-oxabicyclo [9.1.0] dodeca-3,7-diketone	Yu et al. 2018	**102**	1-methyl-7-oxooctyl-2-aldehyde-4,6- dimethoxy benzoic acid	Liu, Zhang et al. 2009
**85**	drimenin	Yu et al. 2018	**103**	aziridine	Liu, Zhang et al. 2009
**86**	pentadecanoic acid	Lin et al. 2012	**104**	hydrochloride	Liu, Zhang et al. 2009
**87**	1,1,4a-trimethyl-5,6-dimethylenedecahydronaphthalen	Lin et al. 2012	**105**	2-[4-dimethylaniline]-3-hydroxy-4-H-chromene-4-ket	Liu, Zhang et al. 2009
**88**	5,5-dimethyl-4-(3-methyl-1,3-butenyl)-1-oxaspiro-[2.5]-ooctane	Li 2007	**106**	(1*R*,3a*S*,5a*S*,8a*R*)-1,3a,5a-trimethyl-4-methylenedecahydropenta	Lin et al. 2012
**89**	(*E*,*E*)-6,10,14-trimethylpentadeca-5,9,13-trien-2-one	Lin et al. 2012; Yao et al. 1999	**107**	2-methyl-5-amino-3,3,4-trinitrile-2,3-dihydrofuran	Liu, Zhang et al. 2009
**90**	3-hexen-1-ol	Lin et al. 2012; Yao et al. 1999	**108**	ocimene	Lin et al. 2012; Yao et al. 1999
**91**	1-nonene	Lin et al. 2012	**109**	methyl disulfide	Liu, Zhang et al. 2009
**92**	1-octen-3-ol	Lin et al. 2012	**110**	dichlone-2-nitropropane	Liu, Zhang et al. 2009
**93**	6-methyl-5-hepten-2-one	Lin et al. 2012	**111**	3-phenyl-1,3-oxyzazcyclo-2-butanone	Liu, Zhang et al. 2009
**94**	myrene	Lin et al. 2012	**112**	methylamine	Liu, Zhang et al. 2009
**95**	dl-6-methyl-5-hepten-2-ol	Lin et al. 2012	**113**	thiophene(3,2-*e*) benzofuran	Liu, Zhang et al. 2009
**96**	acetate	Lin et al. 2012	**114**	5-hydroxy-4-octanone	Liu, Zhang et al. 2009
**97**	*N*-(2-ethylamine)-ethylenimine	Liu, Zhang et al. 2009	**115**	2,3-dimethyl-epoxy-2-methylpropane	Liu, Zhang et al. 2009
**98**	undecane	Lin et al. 2012	**116**	di-undecyl phosphate	Liu, Zhang et al. 2009
**99**	decanal	Lin et al. 2012	**117**	α-naphtho	Liu, Zhang et al. 2009
**118**	decanol	Lin et al. 2012	**140**	2-cyclohexyl-4,6-dinitrophenol	Liu, Zhang et al. 2009
**119**	indole	Lin et al. 2012	**141**	1-propoxy-pentane	Liu, Zhang et al. 2009
**120**	undecanal	Lin et al. 2012	**142**	methyl vinyl ketone	Liu, Zhang et al. 2009
**121**	decanoic acid menthyl ester	Lin et al. 2012	**143**	2-methyl-2-tert butyl-1,3-dithiane	Liu, Zhang et al. 2009
**122**	1-undecanol	Lin et al. 2012	**144**	3-hexen-1-ol	Liu, Zhang et al. 2009
**123**	2,3-diethyl-1,3-heptadiene	Lin et al. 2012	**145**	β-propiolactone	Liu, Zhang et al. 2009
**124**	zingiberene	Lin et al. 2012; Yao et al. 1999	**146**	elemene	Lin et al. 2012; Yao et al. 1999
**125**	ethyh caprate	Lin et al. 2012	**147**	4-butanediol acrylate	Liu, Zhang et al. 2009
**126**	dodecanal	Lin et al. 2012	**148**	triisopropy-trioxane	Liu, Zhang et al. 2009
**127**	α-bergamotene	Lin et al. 2012	**149**	diazirin-ethylamine	Liu, Zhang et al. 2009
**128**	2,4,6 tripropyl-1,3,5-trioxane	Liu, Zhang et al. 2009	**150**	1-cyclobutane cyclobutene	Liu, Zhang et al. 2009
**129**	α-humulene	Lin et al. 2012; Yao et al. 1999	**151**	3,5-dimethy-3-hydrogenation-2-thione-1,3,4-thiadiazoles	Liu, Zhang et al. 2009
**130**	*trans*-β-farnesene	Lin et al. 2012	**152**	diallyl disulfide	Liu, Zhang et al. 2009
**131**	1-dodecanol	Lin et al. 2012	**153**	dibutylene dicyanide	Liu, Zhang et al. 2009
**132**	1-decene	Lin et al. 2012	**154**	ethyl nitrite	Liu, Zhang et al. 2009
**133**	hexahydro-1,3,5-trinitro-1,3,5-acetanilide	Liu, Zhang et al. 2009	**155**	1,4-butanedio-butly etherr	Liu, Zhang et al. 2009
**134**	1-tetradecanol	Lin et al. 2012	**156**	ethyl-oxirane	Liu, Zhang et al. 2009
**135**	*N*-(3-methyl-dibromo) phenylethylamine	Liu, Zhang et al. 2009	**157**	10-heptadecen-8-ynoic acid, methyl ester	Wu et al. 2007
**136**	methyl laurate	Lin et al. 2012	**158**	zeaxanthin	Wu et al. 2007
**137**	santalol	Lin et al. 2012	**159**	cyclopropane	Liu, Zhang et al. 2009
**138**	farnesol	Lin et al. 2012	**160**	2-allylphenol	Lin et al. 2012
**139**	*N*-methyl sulfone-imidazole	Liu, Zhang et al. 2009	**161**	cinerone	Wang et al. 2017
**162**	octahydro-8,8a-dimethyl-2(1H)- naphthalenone	Wu et al. 2007	**177**	2,6,6-trimethyl-1-cyclohexene-1-propyl alcohol	Wu et al. 2007
**163**	ethyl laurate	Lin et al. 2012	**178**	16-octadecenal	Lin et al. 2012
**164**	4-(2,6,6-trimethyl-2- cyclohexene-1-xy)-2-butanone	Wu et al. 2007	**179**	2-methyl-4-(2,6,6-trimethyl cyclohexene) butylene-2-al-1-ol	Wu et al. 2007
**165**	9,11-dimethyltetracyclo-[7.3.1.0(2.7).1(7.11)]-tetradecane	Wang et al. 2017	**180**	2-H-pyran,2-(7-heptadecynyloxy)-tetrahydro-1,4-methanoazulen-3-ol	Wu et al. 2007
**166**	butyl-2-methyl butyrate	Lin et al. 2012	**181**	xanthoxylin	Wu et al. 2007
**167**	10,10-dimethyl-2,6-dimethylenebicyclo [7,2,0] undecane-5-ol	Lin et al. 2012	**182**	junipene	Lin et al. 2012; Wang et al. 2017
**168**	elsholtzia ketone	Wang et al. 2017	**183**	2-vinylnaphthalene	Wang et al. 2017
**169**	(3*E*)-3methyl-4-(2,6,6-trimethylcyclohex-2-en-l-yl) but-3-en-2-ol	Lin et al. 2012	**184**	1,2,3,4-tetrahydro-1,1,6-trimethylnaphthalene	Wang et al. 2017
**170**	neral	Lin et al. 2012; Yao et al. 1999	**185**	9-desoxo-9-x-acetoxy-3,8,12-tri-*O*-acetylingol	Wu et al. 2007
**171**	cycloheptane-1,3,6-trislmethylene	Lin et al. 2012	**186**	1-methylaziridine	Liu, Zhang et al. 2009
**172**	α-longipinene	Lin et al. 2012	**187**	1,3,8-*p*-menthatriene	Lin et al. 2012
**173**	2,6-dimethyl-2,4-heptadiene	Lin et al. 2012	**188**	1-(1H-imidazol-4-yl)-1-pentanone	Wang et al. 2017
**174**	2,3,4,5- tetramethyl-tricyclic-[3.2.1.02,7]-3- caprylene	Wu et al. 2007	**189**	3,7,7-trimethyl-1-(3-oxo-but-1-enyl)-2-oxa-bicyclo-[3.2.0]-hept-3-en-6-one	Wang et al. 2017
**175**	3,5,6,7,8,8a-hexahydride-4,8a-dimethy-6-(1-methylethylidene)- 2(1H)- naphthalenone	Li 2007	**190**	1,4,5,6,7,7a-piperazidine-4-methyl-7-(2-methyl ethyl)-2H-indene-2-ket	Li 2007
**176**	4,6-quinolinediamine	Wang et al. 2017	**191**	6,10,14-trimethyl-2-pentadecacone	Wang et al. 2017
**192**	docosane	Lin et al. 2012	**206**	bisabolene epoxide	Wang et al. 2017
**193**	tricosane	Lin et al. 2012	**207**	phenanthrene	Wang et al. 2017
**194**	benzaldehyde	Yao et al. 1999	**208**	mustardseed oil	Yao et al. 1999
**195**	benzyl alcohol	Yao et al. 1999	**209**	methyl(*Z*)-hexadec-9-enoate	Wang et al. 2017
**196**	benzene acetaldehyde	Yao et al. 1999	**210**	3-butyn-2-yl cyclohexylmethyl phthalate	Wang et al. 2017
**197**	6,10-dimethyl-5,9-undecadiene-2-one	Li 2007	**211**	hexadecenoic acid ethyl ester	Wang et al. 2017
**198**	hexadecenoic acid methyl ester	Wang et al. 2017	**212**	ethyl-9-hexadecenoate	Wang et al. 2017
**199**	farnesene	Yao et al. 1999	**213**	benzene ethanol	Yao et al. 1999
**200**	9-octadecenoic acid	Yao et al. 1999	**214**	linoleic acid	Wang et al. 2017
**201**	spiro [2,4] heptanes 1,5-dimethyl-6-methylene	Lin et al. 2012	**215**	5H-pyrano-[4,3-*b*]-pyridine −3-carbonitrile	Wang et al. 2017
**202**	1-isopropenyl-2-methyl-benzene	Yao et al. 1999	**216**	9-hexadecenoic acid octadecyl ester	Wang et al. 2017
**203**	*trans*-phytoene-4a,7,7-trimethyl-2(1H)- naphthalenone	Li 2007	**217**	9,12-octadecadienoic acid	Yao et al. 1999
**204**	9-eicosyne	Yao et al. 1999	**218**	decanoyl acetaldehyde	Li 2007
**205**	9,12-octadecadienoic acid methyl ester	Yao et al. 1999			

### Terpenoids

**Figure 5.**
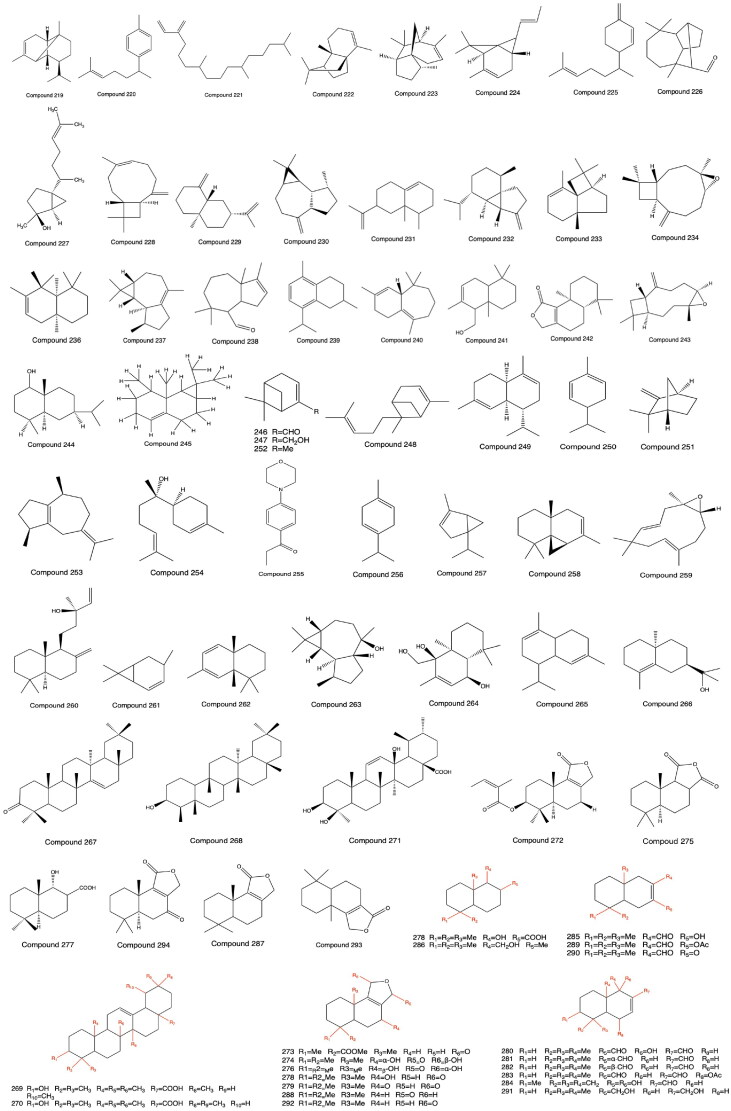
Structures of Compounds **219** to *293*.

The terpenoid compounds of PH are almost all monoterpenes and triterpenes, including α-copaene (**219**), curcumene (**220**), neophytadiene (**221**), 8-(3-methy-2-butanol)-tricyclene (**222**), cedrene (**223**), 8-(3-methyl-2-butenyl)-α-pinene (**224**), β-sesquiphellandrene (**225**), longifolene aldehyde (**226**), 7-*epi*-*cis*-sesquisabinene hydrate (**227**), β-caryophyllene (**228**), selinene (**229**), aromadendrene (**230**), eremophilene (**231**), cubebene (**232**), α-panasinsene (**233**), oxide caryophyllene (**234**), chamigrene (**235**), widdrene (**236**), ledene (**237**), 1,5,5,8a-tetramethyl (**238**), 8-isopropyl-2,5-dimethyl-1,2,3,4-tetrahydronaphthalene (**239**), *cis*-himachalene (**240**), drimenol (**241**), naphthol-[1,2-*c*]-furan-1-(3H)-one-4,5,5a,6,7,8,9,9a-octahydro-6,6,9a-trimethy-(-)-drimenin (**242**), caryophyllene oxide (**243**), eudesmol (**244**), aristolene (**245**), myrtanal (**246**), myrtanol (**247**), trans-α-bergmotene (**248**), α-muurolene (**249**), 1-phellandrene (**250**), camphene (**251**), α-pinene (**252**), guaiene (**253**), α-bisabolol (**254**), elemol (**255**), γ-terpinene (**256**), α-thujene (**257**), thujopsene (**258**), humulene epoxide II (**259**), 1-naphthalenepropanol (**260**), *trans*-carene (**261**), thujopsene-I3 (**262**), globulol (**263**), 1,4,4α,5,6,7,8,8a-octahydro-2,5,5,8α-tetramethyl-β-eudesmol (**264**), 1,2,4a,5,6,8a-hexahydro-4,7-dimethyl-1(1-methylethyl) naphthalene (**265**), 10-*epi*-γ-eudesmol (**266**), taraxerone (**267**), friedelinol (**268**), ursolic acid (**269**), oleanolic acid (**270**), 3β,13β-dihydroxyl-11-ene-28-ursolic acid (**271**), 3β-angeloyloxy-7-epifutronolide (**272**), polygonumate (**273**), dendocarbin L (**274**), (+) winterin (**275**), (+) fuegin (**276**), changweikangic acid A (**277**), futronolide (**278**), 7-ketoisodrimenin (**279**), warburganal (**280**), polygodial (**281**), isopolygodial (**282**), ugandensidal (**283**), muzigadial (**284**), polygonal (**285**), drimenol (**286**), isodrimeninol (**287**), octylene (**288**), monoacetate (**289**), α,β,β′-disubstituted furano (**290**), drimanediol (**291**), isodrimenin (**292**), and confertifolin (**293**) (Fukuyama et al. 1980, 1985; Yao et al. 1999; Zhang and Zeng 2005; Li 2007; Wu et al. 2007; Huang et al. 2012; Lin et al. 2012; Goswami et al. 2014; Wang et al. 2017; Xu et al. 2017; Yu et al. 2018). The structures from **219** to **293** are shown in Figure 5.

### Organic acids

**Figure 6.**
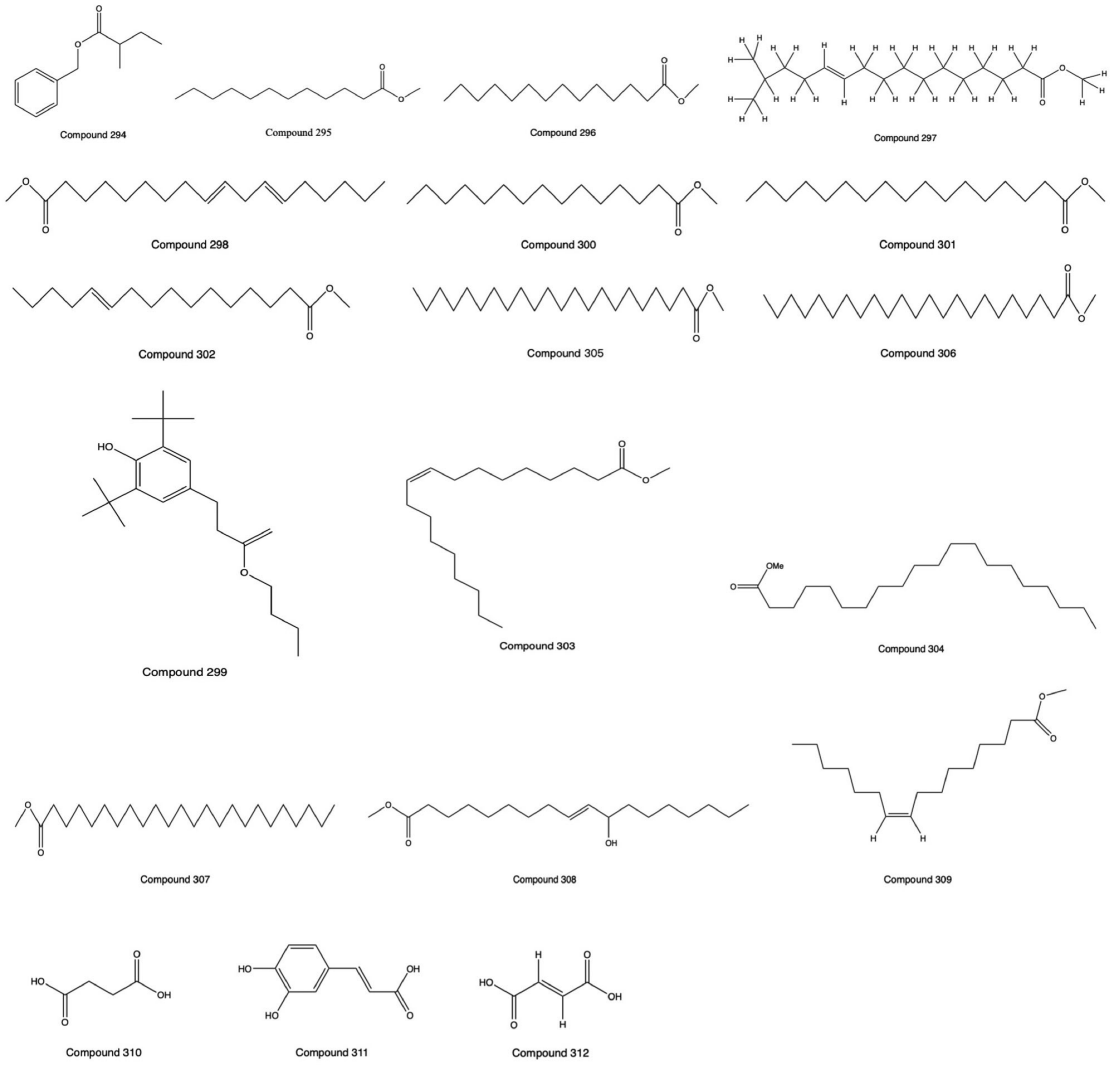
Structures of Compounds **294** to **312**.

Multiple compounds of the organic acid are found in PH, such as fatty acids, polyphenols and carboxylic acids. The fatty acid-like components are mostly unsaturated fatty acids. Sixteen constituents of organic acids were isolated and identified by GC–MS (Liu, Qin et al. 2009), and 3 organic acids were purified and isolated by column chromatography (Li et al. 2017; Xu et al. 2017). The compounds and molecular formulas from **309** to **327** are shown in Table 3. The structures from **294** to **312** are shown in Figure 6.

**Table 3.** Compounds and molecular formulas of organic acids.

No.	Compound	Ref.	No.	Compound	Ref.
**294**	2-methyl-butanoic acid methyl ester	Liu, Qin et al. 2009	**304**	eicosanoic acid methyl ester	Liu, Qin et al. 2009
**295**	dodecanoic acid methyl ester	Liu, Qin et al. 2009	**305**	docosanoic acid methyl ester	Liu, Qin et al. 2009
**296**	methyl tetradecanoate	Liu, Qin et al. 2009	**306**	tricosanoic acid methyl ester	Liu, Qin et al. 2009
**297**	15-methyl-11-hexadecenoic acid methyl ester	Liu, Qin et al. 2009	**307**	tetracosanoic acid methyl ester	Liu, Qin et al. 2009
**298**	9,12-octadecadienoic acid methyl ester	Liu, Qin et al. 2009	**308**	9-octadecenoic acid methyl ester	Liu, Qin et al. 2009
**299**	3,5-*bis*(1,1-dimethylethyl)4-hydroxy-benzenpropanoic acid methyl eater	Liu, Qin et al. 2009	**309**	9-hexadecenoic acid methyl ester	Liu, Qin et al. 2009
**300**	pentadecanoic acid methyl ester	Liu, Qin et al. 2009	**310**	succinic acid	Xu et al. 2017
**301**	heptadecanoic acid methyl ester	Liu, Qin et al. 2009	**311**	caffeic acid	Xu et al. 2017
**302**	hexadecenoic acid methyl ester	Liu, Qin et al. 2009	**312**	fumaric acid	Li et al. 2017
**303**	octadecanoic acid methyl ester	Liu, Qin et al. 2009			

### Steroids

**Figure 7.**
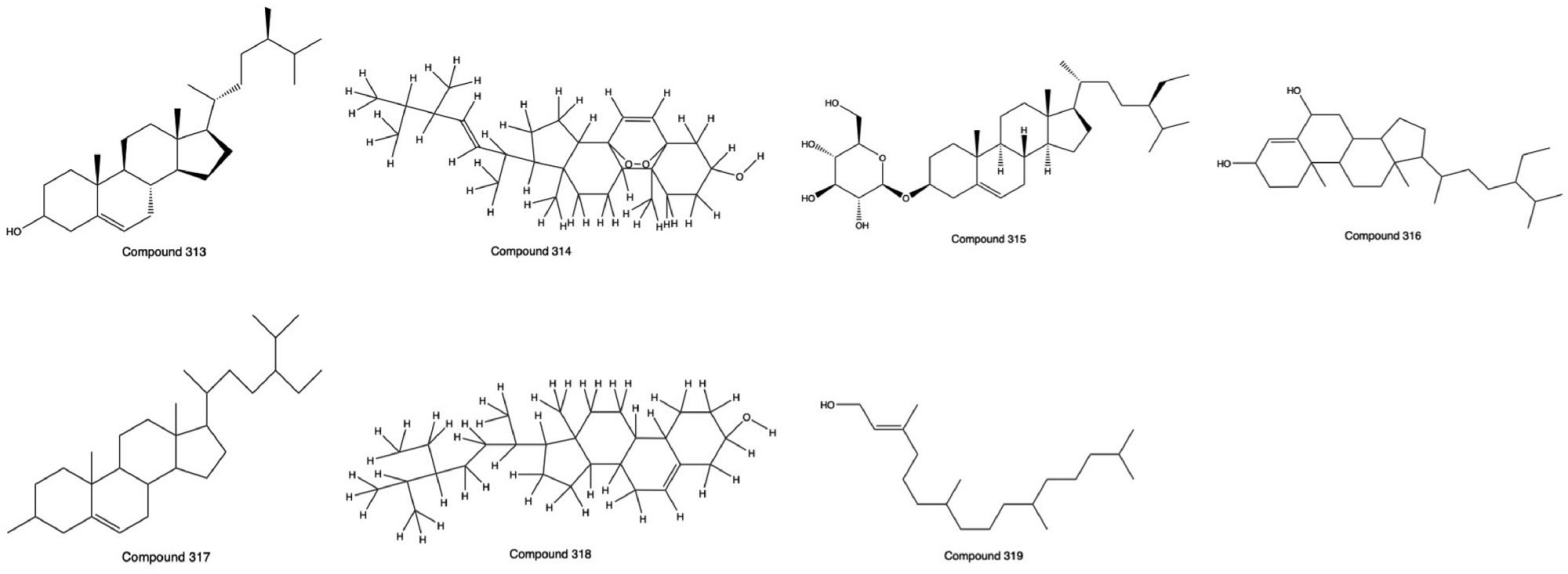
Structures of Compounds **313** to **324**.

A variety of sterols and phytosterols exist in PHL. At present, 7 steroid compounds have been isolated and identified independently, including β-sitosterol (**313**), ergosterol-5,8-peroxide (**314**), daucosterol (**315**), stigmast-4-ene-3β,6a-diol (**316**), γ-sitosterol (**317**), 22,23-dihydrostigmasterol (**318**) and phytol (**319**) (Liu, Qin et al. 2009; Li et al. 2017; Wang et al. 2018). The structures from **313** to **319** are shown in Figure 7.

### Others

**Figure 8.**
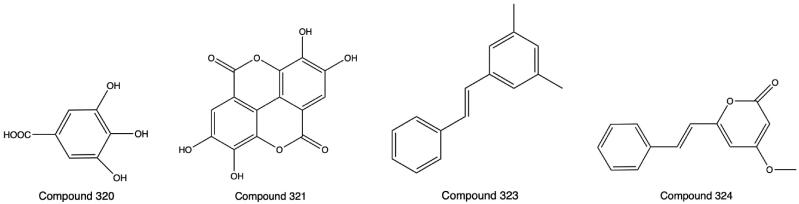
Structures of Compounds **320**, **321**,**323** and **324**.

Gallic acid (**320**), which is the tannin monomer, was isolated from PH extract with 75% ethanol (Huang et al. 2012). Ellagic acid (**321**), a tannin component, was obtained from PH (Li et al. 2017). An acidic polysaccharide named PFMP (**322**) was separated by DEAE column chromatography and authenticated to consist of d-mannose, l-rhamnose, d-glucuronic acid, d-galactose, d-glucose, and l-arabinose by HPLC (Zhu 2020). Pinosylvin (**323**) and 5,6-dehydrokawain (**324**) were isolated from chloroform extract (Xiao 2018). Rich metallic elements, such as Ca, Mg, Al, K, Fe, Mn, Ag, and Zn, and several harmful metals, such as Pb, As, Cu, Hg, and Cd, were discovered by microscopic with identification combined inductively coupled emission spectrometry (Wang et al. 2019). The contents of four heavy metals, Pb, Cd, As, and Hg, were detected by flame atomic absorption spectroscopy, and all met the Green Trade Standard (Lai et al. 2011). The structures of **320**, **321**, **323** and **324** are shown in Figure 8.

## Pharmacological activities

### Antibacterial and antifungal effects

The extracts from different parts of PH have some antibacterial and antifungal effects, especially *in vitro*, showing more extensive antibacterial and antifungal effects. The components of PH, including volatiles, flavones and carboxylic acids, have good broad-spectrum inhibition of bacterial activity (Lin et al. 2012; Li et al. 2017; Ma et al. 2017). The ethanol extract and acetone extract of PH have antifungal activity and may be used in the treatment of fungal infections, such as *Trichospora photospora* and *Pestalotia funerea* in poultry. In conclusion, the active ingredients of PH with antibacterial and antifungal activities may be flavonoids and volatile oils. The active fractions and antibacterial and antifungal species of PH with antibacterial and antifungal activities are shown in Table 4.

**Table 4.** Active fractions with antibacterial and antifungal activity, and antibacterial and antifungal species.

Active compositions	Species	Ref.
flavone ingredients (especially quercetin)	*Staphylococcus* from bacteria	Shi et al. 2017
volatile oil constituents	*Staphylococcus citreus*, *Bacillus paratyphosus*, beta hemolytic *Streptococcus*, F's dysentery *Bacillus*, dysentery *Bacillus* -- from bacteria	Wang et al. 2017
the ethyl acetate portion	*In vivo*: *Staphylococcus aureus*, methicillin-resistant *Staphylococcus aureus* (MRSA)and β -lactamase positive *Staphylococcus* *aureus* -- from bacteria *In vitro*: enteropathogenic *Escherichia coli* -- from bacteria	Wang et al. 2013; Luo, Cheng et al. 2017; Luo 2017
the concentration extract above 0.6 g/mL	*Staphylococcus aureus*>*E. coli*> *Bacillus subtilis* (from bacteria)	Lin 2011
compound extract of the main ingredient	*E. Coli* -- from bacteria	Chen et al. 2012
alcohol extract	*Trichospora photospora* -- from fungus	Zhang et al. 2021
alcohol extract	*Pestalotia funereal* -- from fungus	Zhang et al. 2020
ethanol and acetone extracts	*Candida albicans* -- from fungus	Liu et al. 2012

### Antiviral effects

Both the ethyl acetate and *n*-butanol portions of PH have antiviral effects. The main active component with antiviral effects is flavonoids (Zhou et al. 2020; Lu et al. 2021). The antiviral species and mechanisms of PH are shown in Table 5.

**Table 5.** Antiviral species and mechanisms of PH.

Active compositions	Antiviral species	Mechanisms	Ref.
the ethyl acetate portion	Anti-porcine pseudorabies virus	Inhibited virus proliferation, directly inactivated viruses, and produced antiviral effects, with dose-dependent manner.	Lu et al. 2021
the n-butanol portion	Anti-porcine reproduction and anti-respiratory syndrome virus *in vitro*	Inhibit the synthesis and release of virus, and directly kill the virus to control the proliferation of virus on cells.	Zhou et al. 2020

### Antifeedant and insecticide effects

Because PH has strong antifeedant and insecticidal effects, it could be used in the development of novel pesticides and insecticides in the future. The active constituents of PH with antifeedant and insecticidal effects may be terpenoids. The active ingredients of PH with antifeedant or insecticidal and insect categories are shown in Table 6.

**Table 6.** The active ingredients and insect categories of PH with antifeeding and insecticidal effects.

Active ingredients	Activities	Insect categories	Ref.
total extracts	antifeedant effect and contact toxicity	Cone worm, oblique wing germanica, *Prodenia litura* and cabbage caterpillar	Xiao 2018; Tripathi et al. 1999
water extract	killing effects	Pale culex mosquitoes	Cao 2006
alcohol extract	contact toxicity	*Tetranychus cinnabarinus*	Cheng et al. 2014
ether extract from 95% ethanol extract	contact toxicity	*Pieris rapae* Linne	Xiao 2018; Tripathi et al. 1999
95% ethanol extract	contact toxicity and antifeedant effect	*Ectropis obliqua* Prouy	Chen et al. 2007
ethyl acetate portion	insect-resistant activity	Plasmodial, *Trypanosoma brucei*	Wang et al. 2018
volatile oil constituents	killing effect	*Plutella xylostella*, aphidocolin	Li 2007
Eugenol	antifeedant effect and contact toxicity	*Ectropis obliqua hypulina* Wehrli	Zeng 2007
confertifolin (the essential oil compound from PHL)	killing effect	Mosqutioes, *Anopheles stephensi* and *Culex quinquefasciatus*	Maheswaran and Ignacimuthu 2013

### Antioxidant activity

The flavonoids from PH have good antioxidant activity, and the antioxidant activity of PH is different because of its different structure. Flavonoids with more enol structures have stronger antioxidant capacity, and quercetin has stronger antioxidant activity than vitamin C (Li et al. 2017; Ma et al. 2017). The alcohol extracts from different parts of PH have the ability to scavenge DPPH in the order of caffeic acid > rutin > PH flowers > PH leaves > PH stems > PH roots and the ability to scavenge OH^-^ in the order of PH leaves > PH stems of > PH roots > PH flowers > caffeic acid > rutin (Yang et al. 2014). Muhammad et al. (2014) found that *n*-hexane, chloroform, ethyl acetate, *n*-butanol, water and saponins of PH all had certain antioxidant activities, which were all concentration-dependent on DPPH and could inhibit the activity of acetylcholinesterase. The hexane, chloroform, ethyl acetate, and water fractions of PH had inhibitory activity on butyrylcholinesterase. Yagi et al. (1994) determined the antioxidant activity of flavonoids in PH by the ferric thiocyanate method and discovered that PH had a high eradication rate of superoxide ions in a dose-dependent manner. It was speculated that flavonoids might play a role in scavenging superoxide anions by inhibiting xanthine and xanthine oxidase, thus having antioxidant activity. Sharif et al. (2013) discovered that methanol, ethanol, chloroform, petroleum ether and *n*-hexane fractions of PH all had antioxidant activity, and methanol, ethanol, and petroleum ether had the strongest activity. Overall, PH has good antioxidant activity and can become a natural antioxidant, probably because it contains a large number of flavonoids, terpenoids, and tannins.

### Anti-inflammatory effects

Both the aqueous and alcoholic extracts of PH herbs have certain anti-inflammatory effects. Their active ingredients and their anti-inflammatory effects, inflammation categories, inflammatory modeling methods and anti-inflammatory mechanisms or pathways are shown in Table 7.

**Table 7.** Anti-inflammatory active ingredients, dosage, mode of administration, inflammatory categories, effects, modeling methods and anti-inflammatory mechanisms or pathways of PH.

Ingredients, dosage, and mode of administration	Inflammatory categories	Effects	Modeling methods	Mechanisms or pathways	Ref.
99% methanol extract (100 mg/kg) was administered orally for 7 consecutive days to mice, after modeling. Quercetin was found as one of the active ingredients.	acute colitis	inhibit	Mice were orally administered with 3% dextran sulphate sodium (w/v) in fresh tap water for seven days.	Inhibits the signal pathways of Src/Syk/NF-κB and IRAK/AP-1/CREB.	Yang et al. 2012
Water extract (125, 250, and 500 mg/kg) was administered orally for 7 consecutive days to rats, after modeling.	intestinal inflammation	inhibit	Anus administered rats for 10mg 2,4,6-trinitrobenzenesulfonic acid dissolved in 0.25mL 50% ethanol (v/v) via a 2mm diameter Teflon cannula inserted 8cm into the anus.	500mg/kg water extract treatment significantly ameliorated the activity of MPO and improved the GSH content. There was a downregulation of the TNBS-induced increase in the activity of iNOS and levels of COX-2, TNF-α, and IL-1β while the protein expression of NF-κB was significantly unregulated.	Zhang et al. 2018
N-butanol portion (50, 100, 200 mg/kg) was administered orally for 3 consecutive days to mice before modeling.	sepsis	inhibit	Mice were injected intra-peritoneally with 17 mg/kg (body weight) of E. coli lipopolysaccharide (LPS).	N-butanol portions might contribute to its enhancement in antioxidant capacity, its inhibitory effects may be mediated by inhibiting the phosphorylation of JNK, ERK and c-JUN in MAPKs signaling pathways.	Tao et al. 2016
*n*-butanol portion (50, 100, 150 mg/kg) was administered orally for 5 consecutive days to mice before modeling.	ear swelling in mice	inhibit	Inflammation was induced by applying 0.05 mL of xylene to both sides of the left ear of mice.	N-butanol portions inhibited ear swelling in mice.	Guan et al. 2021
Acetic ether portion (FEA) (40 and 80 μg/mL).	RAW 264.7 cell inflammation model in vitro	relieve	Different doses of acetic ether portion were applied to RAW 264.7 inflammatory response induced by LPS in vitro. and porcine pseudorabies virus	The anti-inflammatory effects of FEA were associated with inhibition of iNOS and COX-2, inhibition of phosphorylation of MAPKs signaling pathway, and increase of expression of phosphorylated AMPK.	Liu et al. 2021
Acetic ether portion (FEA) (40 and 80 μg/mL).	RAW 264.7 cell inflammation model in vitro	relieve	Different doses of FEA were applied to RAW 264.7 inflammatory response induced by Pseudorabies virus in vitro.	Acetic ether portion decreased the secretion of TNF-α, IL-1β, IL-6 and MCP, increased the secretion of IFN-γ, and regulated the secretion of IL-10.	Ren et al. 2021
Acetic ether part of flavone (FEA) and *n*-butanol part of flavone (FNB) (20, 40 and 80 μg/mL).	RAW 264.7 cell inflammation model in vitro	inhibit	In vitro model of inflammation induced by LPS stimulation in RAW 264.7 cells.	FEA and FNB could decrease the release of TNF-α, IL-1β, IL-6 and IL-8 induced by LPS. The anti-inflammatory effect may be related to the anti-oxidative pathway.	Luo, Tao et al. 2017
Acetic ether part of flavone (FEA) (50, 100, 200 mg/kg) were administered orally for 3 consecutive days to mice before modeling.	Endotoxemia	relieve	Mice were injected intra-peritoneally with 17 mg/kg 0.2mL.	The levels of MDA, MPO in intestinal tissue and ACP in serum were decreased in all FEA dosage groups, while the levels of T-AOC, T-SOD, GSH-Px in liver tissue and GSH, LZM in serum were increased in middle and high FEA dosage groups. The levels of TNF-α in serum, intestinal tissue and liver tissue were significantly decreased in all FEA dosage groups, and the mRNA expressions of TNF-α, IFN-γ and IL-2 in lung were significantly decreased in all FEA dosage groups.	Gu, Tao, Wu, et al. 2018
*n*-butanol part of flavone (FNB) (50, 100, 200 mg/kg) were administered orally for 3 consecutive days to mice before modeling.	Endotoxemia	relieve	Mice were injected intra-peritoneally with 17 mg/kg 0.2mL.	FNB can reduce the release of pro-inflammatory factors TNF-α, IL-1β, IL-6 and IL-8 induced by LPS stimulation, and reduce the expression level of TNF-α, IFN-α, IFN-γ and IL-2mRNA in lung by enhancing the activity of antioxidant defense enzyme system in mouse liver.	Gu, Tao, Yang, et al. 2018

### Impact on the immune system

PH can inhibit *Escherichia coli* diarrhea and hepatitis B virus to some extent and can resist immunosuppression caused by cyclophosphamide in mice, which proves that it has a certain immunomodulatory effect. The active components of PH with immunomodulatory effects, the impacts on the immune system and the mechanisms of action are shown in Table 8.

**Table 8.** Active components with immunomodulatory effects, dosage, mode of administration, modeling methods, the impact on the immune system and the mechanisms of action.

Active components, dosage, and mode of administration	Modeling methods	Impacts	Mechanisms	Ref.
water extract (5 and 10 g/kg) were orally administered to mice twice daily for 2 consecutive days prior to modeling.	Mice were intraperitoneally injected with E. coli suspension 10 mg/kg once on the first day, the third day and the seventh day respectively.	Protective effect against *E. coli*-induced diarrhea.	Under the condition of E. coli infection, the expression of CYPs mRNA and protein in the duodenum and liver of mice is generally reduced, and PH has obvious regulation effect on CYPs.	Yue 2020
70% ethanol extract (0.05, 0.1, 0.3, 0.6, 0.9, 1.8mg/L).	HepG 2.2.15 cells secrete hepatitis B surface antigen (HBsAg) and hepatitis B E antigen (HBeAg).	Has certain cellular immunity function and anti-hepatitis B virus function *in vitro*, and has a dose-effect relationship.	The 70% ethanol extract of PH could inhibit the secretion of HBsAg and HBeAg by HepG cells in a dose-dependent manner.	Li et al. 2014
*n*-butanol part of flavone (FNB) (25, 50, 100, 150 mg/kg) were administered orally for 7 consecutive days to mice while modeling.	The immunosuppression model was established by interaperitonel injection of 30 mg/kg cyclophosphamide (CTX) for 7 days.	Enhances immunologic function in mice and counteracts CTX-induced immunosuppression in mice.	FNB can alleviate the impacts of immunosuppression by adjusting the level of NO and activities of MPO and NOS.	Xie et al. 2021
Polysaccharides (100, 200 and 400 mg/mL) were administered orally for 10 consecutive days to mice while modeling.	CTX (50 mg/kg) was injected intraperitoneally on the 3rd, 5th, 7th and 9th day.	Has certain immunoregulatory activity.	Polysaccharides promotes macrophage proliferation and phagocytosis in vitro, and protected mice from immunosuppression induced by cyclophosphamide in vivo.	Zhu 2020

### Protection of the gastrointestinal tract

PH aqueous extract has a good therapeutic effect on *E. coli* diarrhea (Xiao et al. 2018), and acute gastric mucosal injury of rats caused by ethanol (Ren et al. 2018). The mechanism of treatment of *E. coli* diarrhea may be related to reducing the release of inflammatory factors in intestinal tissues, improving the intestinal mucosal barrier and intervening with TGF-β/Smad signal transduction. The mechanism of treating acute gastric mucosal injury induced by ethanol may be related to increasing the content of Nrf 2 and the activity of SOD in gastric mucosal tissues (Ayaz, Junaid, Ullah, Sadiq, et al. 2017).

### Others

In addition, PH has analgesic (Sharif et al. 2013), hypoglycemic (Oany et al. 2016), hypotensive (Muhammad et al. 2016; Devarajan 2018), antiangiogenic (Muhammad et al. 2016), antitumor (Muhammad et al. 2016; Ayaz et al. 2019), antifertility and embryo implantation inhibition effects (Daniyal and Akram 2015). PH also has a therapeutic effect on the reproductive system and influences the expression of insulin-like growth factor in the uterus of early pregnancy rats (Goswami et al. 2014). The volatile composition of PH, β-sitosterol, can improve memory deficits and disorders such as Alzheimer’s disease, and is mainly manifested in the aspects of anticholinesterase, improvement of working memory, spontaneous alternating behavior, motor coordination, etc. of transgenic animals (Ayaz et al. 2015, Ayaz, Junaid, Ullah, Subhan, et al. 2017). PH water extracts alleviate hepatic and duodenal injury induced by enteropathogenic E. coli. The mechanism of *E. coli* infection is that PH inhibits the secretion and expression of inflammatory cytokines and regulates the expression level of CYPs (Huang et al. 2022).

## Applications and prospects

### Traditional applications

Many ancient medical books in China described the traditional effects of PH. As recorded in *Mingyi Bielu*: ‘*Polygonum* leaves, the meridian tropism is tongue, could remove the small and large intestinal evil, also benefit in the intelligence.’ As recorded in *Tang Materia Medica*: ‘PH main treatment snake bite, mashed to apply; juice to take, treatment of snake venom caused by abdominal stuffiness; soak feet in water decocted solution of PH, followed by massage, remove beriberi swelling.’ As recorded in *Bencao Shiyi*: ‘PH, the main treatment of periumbilical and hypochondriac firmness and pain, takes 60 g daily after boiling; PH treats cholera with muscular spasm and to massage feet with hot water decoction; the leaves are crushed and applied to treating the huci nevus; the leaves are also used for treating the head sores in children.’ As recorded in *Lingnan Caiyaolu*: ‘PH can be applied to the injury site, can be used to clean the surface of naevus scabies, and also can itch and swell.’ (Zhang 2004).

### Modern applications

PH can be used for preventing and treating enteritis in black carp and grass carp in the high temperature season (July to September every year). The feed method comprises chopped herb PH hay or fresh herb PH decocted with an appropriate amount of water, mixing that filtrate with bait, and continuously feeding for 3-6 days (250 g hay or 1.5 kg fresh herb PH per 50 kg) (Wang et al. 2021). Planting PH on the mud bank of eel ponds can prevent red-skin disease for a long time (Liu 2020). Cleaning and mashing fresh stems and leaves of PH, adding water to soak for 24 h, filtering to remove residues, diluting with water and spraying a solution that is five times that of the original on leaf surfaces helps to prevent and control plant hoppers, aphids, rice leafhoppers, tea caterpillars, etc. The fresh stems and leaves of PH can be washed, dried, powdered and sprinkled to prevent and control cutworms and grubs (Ao 2019; Zhou and Du 2019). Intramuscular injection of the PH liquid can be used for treating porcine epidemic diarrhea (Ren 2017). After drying, PH is crushed and mixed into grain at a ratio of 1:1000, which can keep the grain free of insects for a year. The mung bean weevil can be controlled by putting PH into mung beans at a ratio of 9:1000 (Peng 2016). Feeding dried or fresh PH to fish can prevent and cure rotten gill disease (Wang 2016). PH has been boiled in water and mixed with chopped tender grass or bait to treat fish enteritis (Liu and Wang 2017). PH has also been ground into powder, mixed with bait and fed to cure fish enteritis, rotten gill and red-skin disease (Liu 2017). The stem or whole plant of fresh herba PH can be mashed into mud and applied to the affected part to treat livestock pellagra (Zhang 2014). Clinically, PH and *Gardenia jasminoides* Ellis (Rubiaceae) can treat ovarian cysts (Li and Gu 2018).

There are few studies on the toxicology of PH. At present, only Zhang et al. (2022) and Zhu et al. (2020) found that the flavonoid extract of PH at 5 g/(kg·BW) and below had no acute toxic effects or side effects in mice, has no subchronic toxic effects after long-term continuous medication, and has good safety.

### Prospects

Since PH contains many active ingredients, each with different medicinal effects, it also has more prospects for development and application. The polysaccharide isolated from PH can be used as an immunomodulator in health food. PH can be made into new anti-trypanosomal drugs due to its anti-trypanosomal activity. In places where mosquitoes are very likely to breed, such as cesspools and gutters, a certain amount of volatile oil extract of PH can be added as an insecticide and mite killer. In addition, PH can be made into plant pesticides, antibacterial agents, and natural antioxidants.

## Conclusions

As a traditional herbal medicine with a long history, PH can be used in a single or compound prescription. Because of its multiple effects and medicinal history, many researchers have a keen interest in PH. This review comprehensively summarizes the phytochemical constituents, pharmacological activities, and applications of PH, which could offer ideas and foundations for the further studies.

To date, a total of 324 compounds have been isolated and identified from PH, mainly flavonoids, phenylpropanoids, volatile oils, terpenoids and organic acids. Among them, flavonoids and volatile oil components have certain antibacterial and antiviral effects, but the active ingredients and pharmacodynamic substance basis have not been reported. The volatile oil component ‘eugenol’ was found to be a pesticide, and the insecticidal mechanism was that eugenol significantly inhibited the activities of acetylcholinesterase and glutathione-*S*-transferase (Zeng 2007). The second volatile oil component ‘confertifolin’ was considered for use in the control of human vector mosquitoes, but the mechanism is unclear (Maheswaran and Ignacimuthu 2013). Confertifolin is very promising for formulating a potent and affordable natural product to control dreadful disease transmission and nuisance-creating human vector mosquitoes. The other active insecticidal components were crude extracts. In general, the study of PH remains inadequate. Quercetin is an anti-inflammatory active ingredient, and its mechanism is that it inhibits the Src/Syk/NF-κB and IRAK/AP-1/CREB signaling pathways. It has been suggested that quercetin should be developed as a novel anti-inflammatory remedy (Yang et al. 2012). In particular, there is a lack of toxicity research. To date, only a few studies have reported that the flavonoids of PH have no acute toxicity and are nontoxic after long-term use. However, there is a lack of reports of other active components, especially volatile oil and terpenoid components. When used as pesticides, there is also a lack of reports on the optimal concentration and dose of pesticides for various pests and whether there is harm to the human body when used as pesticides. In ancient China, people often burned PH at night to repel mosquitoes and insects, and this practice could match the insecticidal effect in modern pharmacological research. The polygodial contained in PH is a volatile terpenoid, has good antifeedant activity to insects, and has antioxidation, good stability to air and light, and a half-life of more than one month. It is less stable to heat but has a half-life of more than 15 days, which is different from other herbs (Zhang 2004). While the current study only reported that the active ingredients of PH with insecticidal effects are volatile oils and some crude extracts, the active compounds have not been determined. High-temperature burning has a pungent odor, and whether the insecticidal mechanism is related to the pungent odor is unknown, so the subsequent research should involve more in-depth screening of the active substances with insecticidal effects, which can be developed into pesticides and insecticides of plant origin.

As a common traditional herbal medicine, PH is often grown in ditches and depressions, probably because of its ability to resist the growth of weeds. Currently, with the continuous modernization, an increasing number of skyscrapers are rising from the ground, and the germplasm resources of PH are gradually decreasing. We must pay attention to the conservation of this natural medicinal resource. Since PH grows in gullies and depressions, such places are hotbeds of bacteria and many kinds of microorganisms; however, PH resists the growth of miscellaneous bacteria, probably because it has some important endophytic bacteria of its own. However, no research has been done on this topic yet, which could be another new research direction.
